# Depression and medication adherence among chronic hepatitis B patients receiving resilience-based care versus standard care during antiviral therapy: a retrospective cohort study

**DOI:** 10.3389/fmed.2026.1832041

**Published:** 2026-09-21

**Authors:** Xiao Yang, Ping Zhao, Lei Zhang, Chunhua Fang

**Affiliations:** 1 Department of Infectious Diseases, Hefei Third People’s Hospital, Third Clinical Hospital of Anhui Medical University, Hefei, China; 2 Nursing Department of Hefei Third People’s Hospital, Third Clinical Hospital of Anhui Medical University, Hefei, China

**Keywords:** chronic hepatitis B, depression, resilience intervention, retrospective study, treatment adherence

## Abstract

**Objective:**

To evaluate the association between receiving a resilience-focused program in addition to standard care and depressive symptoms and medication adherence among patients with CHB receiving antiviral therapy.

**Methods:**

In this retrospective cohort study, 200 patients diagnosed with CHB and treated at our hospital's hepatology clinic between July 2022 and June 2025 were included. Based on historical records, 100 patients who voluntarily completed a structured 12-week resilience promotion program in addition to standard care formed the observation group, and 100 patients who received standard care only formed the control group. Primary outcomes at the 6-month follow-up were depressive symptom severity measured by the Patient Health Questionnaire-9 (PHQ-9) and medication adherence measured by the Medication Possession Ratio (MPR). Secondary outcomes included resilience (Connor-Davidson Resilience Scale-10, CD-RISC-10), virological response, alanine aminotransferase (ALT) normalization, quality of life (SF-36), adverse events, and healthcare utilization. To account for potential confounding by indication and baseline imbalances, multivariable linear, logistic, and Poisson regression models were used, with adjusted effect estimates and 95% confidence intervals reported.

**Results:**

At the 6-month follow-up, the observation group had lower depression scores (6.78 ± 3.45 vs. 10.23 ± 4.12), higher MPR (0.92 ± 0.08 vs. 0.78 ± 0.15), and higher resilience scores (32.45 ± 5.67 vs. 26.78 ± 6.34). Virological response (85.0% vs. 70.0%; adjusted OR 2.46, 95% CI 1.35 to 4.48) and ALT normalization (80.0% vs. 65.0%; adjusted OR 2.01, 95% CI 1.10 to 3.67) were more frequent in the observation group. SF-36 physical and mental component scores were also higher in the observation group. Adverse event incidence was similar between groups. The observation group had fewer unplanned outpatient visits (*P* = 0.045), and Poisson regression confirmed fewer outpatient visits in the observation group (adjusted rate ratio 0.72, 95% CI 0.56 to 0.93). However, because allocation was non-randomized, these associations may be affected by self-selection and residual confounding.

**Conclusion:**

In this retrospective cohort study, receiving a resilience-focused program in addition to standard care was associated with lower depressive symptoms, better medication adherence, and more favorable virological and biochemical outcomes in patients with CHB.

## 1 Introduction

Chronic hepatitis B (CHB) represents a global public health challenge caused by hepatitis B virus (HBV) infection, with long-term persistence potentially leading to severe complications including liver cirrhosis and hepatocellular carcinoma (1). Antiviral therapy constitutes the cornerstone of CHB management; however, extended treatment duration, drug-related side effects, and psychosocial stressors frequently contribute to depressive symptoms and reduced treatment adherence among patients (2). Depression is highly prevalent in CHB populations, potentially linked to disease-related stress, immune-inflammatory responses, and neuroendocrine dysregulation (3). Suboptimal adherence directly undermines virological suppression, elevating risks of treatment failure and disease progression (4). Psychological interventions, such as cognitive behavioral therapy (CBT) and positive psychology approaches, have been employed in chronic disease management to enhance psychological resilience and self-management capabilities (5). Resilience refers to an individual's capacity to maintain psychological adaptation and positive adjustment in the face of adversity, with levels inversely correlated with depression and positively associated with adherence (6). Previous studies indicate that resilience-based interventions can improve psychological and behavioral outcomes in patients with chronic conditions such as cancer and diabetes; however, evidence within CHB populations remains limited, often focusing on single dimensions such as mood or adherence, and lacking comprehensive multi-dimensional assessment (7).

Although randomized controlled trials provide the strongest evidence for efficacy, their controlled conditions may not fully reflect real-world uptake and outcomes. Observational data from clinical practice can complement these trials by describing implementation patterns and adjusted associations in unselected patient populations (8). Standard care in CHB typically provides medication instructions and brief adherence counseling but lacks structured psychological components to manage disease-related automatic negative thoughts, stigma, and emotional distress—factors associated with non-adherence (9). A resilience-based program targets these cognitive-emotional barriers and may be associated with outcomes not achieved through conventional education alone (10). However, because participation in such programs is typically voluntary in clinical practice, patients who choose to participate may differ systematically from those who do not. This non-random exposure introduces selection bias, confounding by indication, and residual confounding by unmeasured factors such as baseline motivation, social support, and adherence propensity. In the present study, the resilience program was offered to all eligible CHB patients, but uptake was voluntary and depended on patient preference, perceived need, and scheduling availability. These differences cannot be fully captured by measured covariates, and all observed associations should therefore be interpreted cautiously. Several studies in this area have also been limited by short follow-up and incomplete adjustment for baseline clinical and sociodemographic factors (11, 12). Furthermore, biological mechanisms involving the hypothalamic-pituitary-adrenal axis, cortisol, and pro-inflammatory cytokines such as IL-6 and TNF-α have been proposed to link psychological distress with impaired immune control of HBV (13, 14); however, these biomarkers were not measured in the present study, and these pathways remain theoretical.

This study leverages a retrospective cohort design to explore real-world associations between a structured resilience program and multidimensional outcomes in CHB. Although the observational nature precludes causal inference, the inclusion of psychological, behavioral, virological, and healthcare utilization endpoints, together with multivariable adjustment, allows a more integrated description of potential benefits and limitations of such interventions. The findings are intended to generate hypotheses and inform the design of future prospective trials, rather than to validate intervention efficacy.

## 2 Materials and methods

### 2.1 Study population

This retrospective cohort study utilized data extracted from the electronic medical record system of our hospital's hepatology clinic. A total of 200 patients diagnosed with chronic hepatitis B (CHB) and receiving antiviral therapy between July 2022 to June 2025 were included. Based on historical records, 100 patients who voluntarily and regularly completed a systematic “Resilience Promotion Program” in addition to routine treatment were assigned to the observation group; the remaining 100 patients received standard care only and were designated as the control group. Baseline demographic and clinical characteristics of both groups, retrieved and summarized in Table 1 (presented in the Results section), indicated that the group distribution reflected the natural proportion of eligible patients receiving structured psychological intervention in real-world practice. The observation group comprised 59 males and 41 females, with a mean age of 44.01 ± 12.32 years; the control group included 66 males and 34 females, with a mean age of 46.53 ± 13.97 years. No statistically significant intergroup differences were observed at baseline (*P* > 0.05).

**Table 1:** Patient screening and enrollment process.

Screening step	Number	Reason/detail
Initial screening	412	Patients with CHB assessed for eligibility
Excluded: other liver diseases	68	HCV, alcoholic liver disease, NASH
Excluded: missing key data	63	>20% missing records
Excluded: prior psychotherapy	47	Received psychotherapy/pharmacotherapy within 6 months
Excluded: initiated but did not complete resilience program	34	Started but completed <12 sessions
Final enrollment	200	Observation group (*n* = 100), Control group (*n* = 100)

No prospective sample size calculation was performed because this retrospective study included all eligible patients during the study period. A *post hoc* power analysis was not used for the primary interpretation, because observed power is a function of the *P* value and does not add information beyond confidence intervals. The Resilience Promotion Program was an optional, clinic-based service offered to all CHB patients; participation was voluntary and not part of standard medical care. The PHQ-9 was administered to all patients as part of routine psychosocial screening during nurse-led education sessions. Among patients who initiated the program, 34 did not complete all 12 sessions and were excluded from the observation group to minimize completer bias; this exclusion is shown in the participant flow diagram (Table 1). Nevertheless, self-selection and residual confounding remain possible.

### 2.2 Inclusion and exclusion criteria

The following criteria were applied to ensure population homogeneity and outcome comparability:

Inclusion Criteria:

1. Age between 18 and 70 years;
2. Diagnosis of CHB according to the Guidelines for the Prevention and Treatment of Chronic Hepatitis B, and continuous antiviral therapy with nucleos(t)ide analogues (e.g., entecavir or tenofovir) for ≥12 months;
3. Availability of complete baseline and follow-up (6 months post-intervention) records for psychological assessments and laboratory tests;
4. For the observation group, documented participation in and completion of all sessions of the “Resilience Promotion Program.”

Exclusion Criteria:

1. Coexisting liver diseases of other etiologies, such as hepatitis C virus (HCV) infection, alcoholic liver disease, or non-alcoholic steatohepatitis;
2. History of decompensated cirrhosis, hepatocellular carcinoma, or liver transplantation;
3. Prior diagnosis of severe psychiatric disorders such as schizophrenia or bipolar disorder, or receipt of professional psychotherapy or pharmacotherapy for depression within 6 months prior to baseline;
4. Pregnancy or lactation;
5. Missing key information in medical records exceeding 20%.

### 2.3 Study methods

#### 2.3.1 Study design and data extraction process

This study adhered to the Strengthening the Reporting of Observational Studies in Epidemiology (STROBE) guidelines. Data extraction was independently performed by two research assistants blinded to group allocation and not involved in clinical management. A predefined data collection form was used to extract the following from electronic medical records: demographic information, clinical history, antiviral treatment regimen, laboratory results, and all assessment records corresponding to the observed outcomes. Any discrepancies in extracted data were resolved by a third senior researcher through review of original records.

#### 2.3.2 Definition of exposure and intervention details

The primary exposure variable was “receipt of systematic resilience intervention.” The “Resilience Promotion Program” administered to the observation group was a structured group intervention based on cognitive behavioral therapy (CBT) and positive psychology principles, designed to enhance patients’ intrinsic capacity to cope with disease-related stress. The program spanned 12 weeks, with one 90-minute session per week, co-facilitated by a certified clinical psychologist and a hepatology nurse specialist. Intervention modules included: (i) Psychoeducation: covering CHB disease knowledge, treatment goals, and the importance of long-term management, while addressing misconceptions; (ii) Cognitive Restructuring: identifying disease-related automatic negative thoughts (e.g., “the disease is incurable,” “I am a burden to my family”) and techniques to challenge and replace them; (iii) Stress and Emotion Management: training in relaxation techniques (e.g., progressive muscle relaxation), mindful breathing, and emotional labeling; (iv) Problem-Solving Skills Training: role-playing and discussion of solutions to adherence barriers (e.g., forgetfulness, side effect concerns); (v) Resilience Resource Building: guiding patients to explore and strengthen personal strengths, sense of value, and social support networks. The control group received standard care, comprising disease education, medication guidance, and necessary medical advice provided by attending physicians during routine follow-ups, without the structured, periodic psychological skill-building sessions.

Temporal definitions. Baseline (index date) was defined as the date of the first psychosocial screening record during the study period. For the observation group, the intervention period was the subsequent 12 weeks, and the 6-month follow-up outcome assessment was performed at 6 months after the index date (i.e., approximately 3 months after program completion). For the control group, there was no intervention period, and the 6-month follow-up outcome assessment was also performed at 6 months after the index date. Baseline PHQ-9, CD-RISC-10, SF-36, HBV DNA, and ALT values were incorporated into adjusted follow-up models using analysis of covariance (ANCOVA) for continuous outcomes.

#### 2.3.3 Bias control strategies

Because participation in the resilience program was voluntary, self-selection bias, confounding by indication, and completer bias are fundamental limitations. Patients who chose the intervention and completed all sessions may have had higher baseline motivation, better social support, and a greater propensity for adherence, which cannot be fully captured by measured covariates. To quantify baseline comparability, we calculated standardized differences for all baseline covariates; standardized differences <0.10 were considered negligible, and any covariates with standardized differences ≥0.10 were considered for adjustment regardless of *P* value. All adjusted estimates should be interpreted as associations conditional on observed variables, not as unbiased causal effects.

Fibrosis stage was not systematically recorded; cirrhosis was identified from clinical documentation based on ultrasonography, transient elastography (where available), or FIB-4/APRI indices. Detailed antiviral treatment history (prior switches, interruptions) and quantitative HBsAg levels were not uniformly available in the electronic records and thus could not be included. Baseline adherence patterns before the index period were not formally measured.

### 2.4 Outcome measures

#### 2.4.1 Severity of depressive symptoms

Assessed using the Patient Health Questionnaire-9 (PHQ-9). This 9-item instrument is scored from 0 (not at all) to 3 (nearly every day) per item, yielding a total score ranging from 0 to 27. Interpretation: 0–4 (none), 5–9 (mild), 10–14 (moderate), 15–19 (moderately severe), 20–27 (severe). Administered at baseline and 6 months post-intervention. PHQ-9 was administered by trained nurses as part of routine psychosocial screening. Patients with PHQ-9 ≥ 15 were offered referral to psychiatry; however, uptake of psychiatric care and use of antidepressants during follow-up were not systematically captured in the hepatology database and represent unmeasured confounders

#### 2.4.2 Medication adherence

Adherence to antiviral therapy was assessed using the Medication Possession Ratio (MPR), calculated from pharmacy refill records within the electronic medical record system. MPR was defined as the total days’ supply of medication dispensed during the 6-month follow-up period divided by the number of days in the follow-up period (180 days). MPR values range from 0 to 1, with higher values indicating better adherence. MPR was analyzed as a continuous variable, and good adherence was defined *a priori* as MPR ≥ 0.80. Baseline adherence data before the index date were not available; therefore, MPR cannot be interpreted as a change from baseline and is presented only as a follow-up value.

#### 2.4.3 Resilience level

Measured using the Chinese version of the 10-item Connor-Davidson Resilience Scale (CD-RISC-10). This 10-item tool uses a 5-point scale (0–4), with total scores ranging from 0 to 40; higher scores indicate greater resilience. Assessment timing matched that of the PHQ-9.

#### 2.4.4 Virological response

Serum HBV DNA levels were quantified via polymerase chain reaction (PCR). Log10-transformed HBV DNA values (log10 IU/mL) were recorded at baseline and 6 months. Primary virological response was defined as HBV DNA below the lower limit of detection (<20 IU/mL) at follow-up.

#### 2.4.5 Liver inflammation activity

Assessed by serum ALT levels (U/L). ALT values were recorded at baseline and 6 months, and ALT normalization rate was calculated, defined as reduction to within the upper limit of normal (≤35 U/L for men, ≤25 U/L for women). ALT was used as a pragmatic surrogate for hepatic necroinflammation; however, ALT elevations can also result from non-HBV causes (e.g., medications, fatty liver), and normal ALT does not exclude mild histological activity. This limitation is discussed later.

#### 2.4.6 Health-Related quality of life

Evaluated using the Chinese version of the 36-item Short Form Health Survey (SF-36). This instrument comprises 8 domains, summarized into Physical Component Summary (PCS) and Mental Component Summary (MCS) scores; higher scores indicate better quality of life. Assessment timing aligned with the PHQ-9.

#### 2.4.7 Adverse events related to antiviral therapy

Systematically reviewed from patient complaints and physician diagnoses documented in medical records. All adverse events potentially related to antiviral drugs during follow-up (e.g., dizziness, nausea, abnormal renal function indicators) were collected, and overall incidence was calculated.

#### 2.4.8 Healthcare utilization

The number of unplanned outpatient visits and emergency department visits/hospital admissions due to liver-related issues per patient during the 6-month follow-up was recorded, reflecting disease instability and healthcare burden.

### 2.5 Statistical analysis

All analyses were performed using SPSS 26.0 and R 4.3.1, with two-sided tests and a significance level of *P* < 0.05. Primary outcomes were prespecified as PHQ-9 score and MPR at 6 months; all other outcomes were prespecified as secondary. Normally distributed continuous data are presented as mean ± standard deviation (SD), compared using independent samples t-tests; non-normally distributed data are reported as median (interquartile range) [M (IQR)], compared using Mann–Whitney U tests. Categorical data are expressed as frequency (percentage), compared using chi-square or Fisher's exact tests. Standardized differences were calculated for all baseline covariates to assess balance.

For primary and secondary continuous outcomes measured at follow-up (PHQ-9, MPR, CD-RISC-10, SF-36), analysis of covariance (ANCOVA) was used to estimate adjusted between-group differences, with baseline values included as covariates where available. For binary outcomes (virological response, ALT normalization, adverse events, ED/hospitalization), binary logistic regression was used, with adjusted odds ratios (ORs) and 95% confidence intervals (CIs) reported. For healthcare-utilization count data (number of unplanned outpatient visits), we used Poisson regression with robust standard errors as a sensitivity analysis, reporting adjusted rate ratios; the prespecified dichotomized analysis (≥3 visits) was retained for interpretability. For continuous biological indicators measured as change from baseline (HBV DNA and ALT changes), adjusted linear regression models were used. All models included age, sex, body mass index, baseline HBV DNA, baseline ALT, antiviral agent type, and presence of cirrhosis as covariates. Variance inflation factors were <5, indicating no severe multicollinearity. Missing data were minimal (<5% for any variable) and were handled by complete-case analysis; no imputation was performed. Propensity score matching or inverse probability weighting was not employed because the modest sample size and number of potential confounders posed a substantial risk of model overfitting and unstable weights. We refer to all adjusted estimates as “adjusted associations” rather than “effects” because of the potential for residual and unmeasured confounding.

## 3 Results

### 3.1 Patient screening and enrollment process

Table 1 outlines the screening process and final enrollment of study participants. Initial screening identified 412 potentially eligible individuals. After sequential exclusions, a total of 200 patients were ultimately included in the analysis, with 100 assigned to the observation group and 100 to the control group. The primary reasons for exclusion comprised coexisting other liver diseases, missing key follow-up data, and receipt of professional psychotherapy at baseline. This process ensured the homogeneity of the study population and the integrity of the data. See Table 1.

### 3.2 Comparison of baseline clinical and demographic characteristics

Table 2 compares the baseline clinical and demographic characteristics between the two groups. A total of 21 variables were assessed, including age, sex, body mass index (BMI), chronic hepatitis B (CHB) duration, duration of antiviral therapy, baseline hepatitis B virus deoxyribonucleic acid (HBV DNA) level, baseline alanine aminotransferase (ALT) level, type of antiviral agent, hepatitis B e-antigen (HBeAg) status, proportion with liver cirrhosis, smoking history, alcohol history, education level, marital status, employment status, proportion with comorbid hypertension, proportion with comorbid diabetes, baseline Patient Health Questionnaire-9 (PHQ-9) score, and baseline Connor-Davidson Resilience Scale-10 (CD-RISC-10) score. Statistical analyses revealed no significant differences in any of these indicators between the groups (*P* > 0.05), indicating balanced baselines and comparability. See Table 2. Standardized differences for all baseline covariates were <0.10, indicating negligible baseline imbalance. The standardized differences for key variables were: age, 0.19; sex, 0.14; baseline PHQ-9, 0.10; and baseline CD-RISC-10, 0.08. Although some standardized differences exceeded 0.10, all covariates were included in adjusted models regardless of univariate *P* values.

**Table 2:** Comparison of baseline clinical and demographic characteristics.

Characteristic	Observation group (*n* = 100)	Control group (*n* = 100)	Statistic	*P*-value
Age (years)	44.01 ± 12.32	46.53 ± 13.97	t = 1.234	0.218
Male sex	59 (59.0%)	66 (66.0%)	*χ*^2^ = 0.890	0.345
BMI (kg/m^2^)	23.45 ± 3.21	23.18 ± 3.45	t = 0.765	0.445
CHB duration (years)	6.78 ± 4.32	7.23 ± 4.56	t = 1.023	0.307
Antiviral duration (months)	24.56 ± 18.34	26.78 ± 19.23	t = 0.876	0.382
Baseline HBV DNA (log10 IU/mL)	5.67 ± 1.23	5.54 ± 1.34	t = 0.654	0.514
Baseline ALT (U/L)	68.45 ± 45.67	72.34 ± 48.23	t = 1.101	0.272
Treated with Entecavir	65 (65.0%)	62 (62.0%)	χ^2^ = 0.321	0.571
HBeAg positive	38 (38.0%)	42 (42.0%)	χ^2^ = 0.567	0.451
Liver cirrhosis	12 (12.0%)	15 (15.0%)	χ^2^ = 0.211	0.646
Smoking history	25 (25.0%)	28 (28.0%)	χ^2^ = 0.456	0.499
Alcohol history	18 (18.0%)	22 (22.0%)	χ^2^ = 0.389	0.533
Higher education	45 (45.0%)	48 (48.0%)	χ^2^ = 1.234	0.745
Married	78 (78.0%)	75 (75.0%)	χ^2^ = 0.678	0.713
Employed	65 (65.0%)	62 (62.0%)	χ^2^ = 1.012	0.603
Comorbid hypertension	22 (22.0%)	25 (25.0%)	χ^2^ = 0.123	0.726
Comorbid diabetes	15 (15.0%)	18 (18.0%)	χ^2^ = 0.298	0.585
Baseline PHQ-9 score	12.34 ± 4.56	11.89 ± 4.78	t = 0.987	0.325
Baseline CD-RISC-10 score	23.45 ± 6.78	22.89 ± 7.12	t = 1.112	0.267

### 3.3 Comparison of primary psychological and behavioral outcomes at 6 months post-intervention

Table 3 presents the primary psychological and behavioral outcomes at the 6-month follow-up. The PHQ-9 score was lower in the observation group than in the control group (mean difference −3.45; t = 6.42, *P* < 0.001). Medication adherence, as measured by MPR, was higher in the observation group (0.92 ± 0.08) than in the control group (0.78 ± 0.15; t = 8.23, *P* < 0.001). The CD-RISC-10 score was also higher in the observation group (t = 6.67, *P* < 0.001). See Table 3 and Figure 1.

**Table 3:** Comparison of primary psychological and behavioral outcomes at 6 months.

Outcome measure	Observation group (*n* = 100)	Control group (*n* = 100)	Statistic	*P*-value
PHQ-9 score	6.78 ± 3.45	10.23 ± 4.12	T = 6.42	< 0.001
MPR	0.92 ± 0.08	0.78 ± 0.15	T = 8.23	< 0.001
CD-RISC-10 score	32.45 ± 5.67	26.78 ± 6.34	T = 6.67	< 0.001

**Figure 1:**
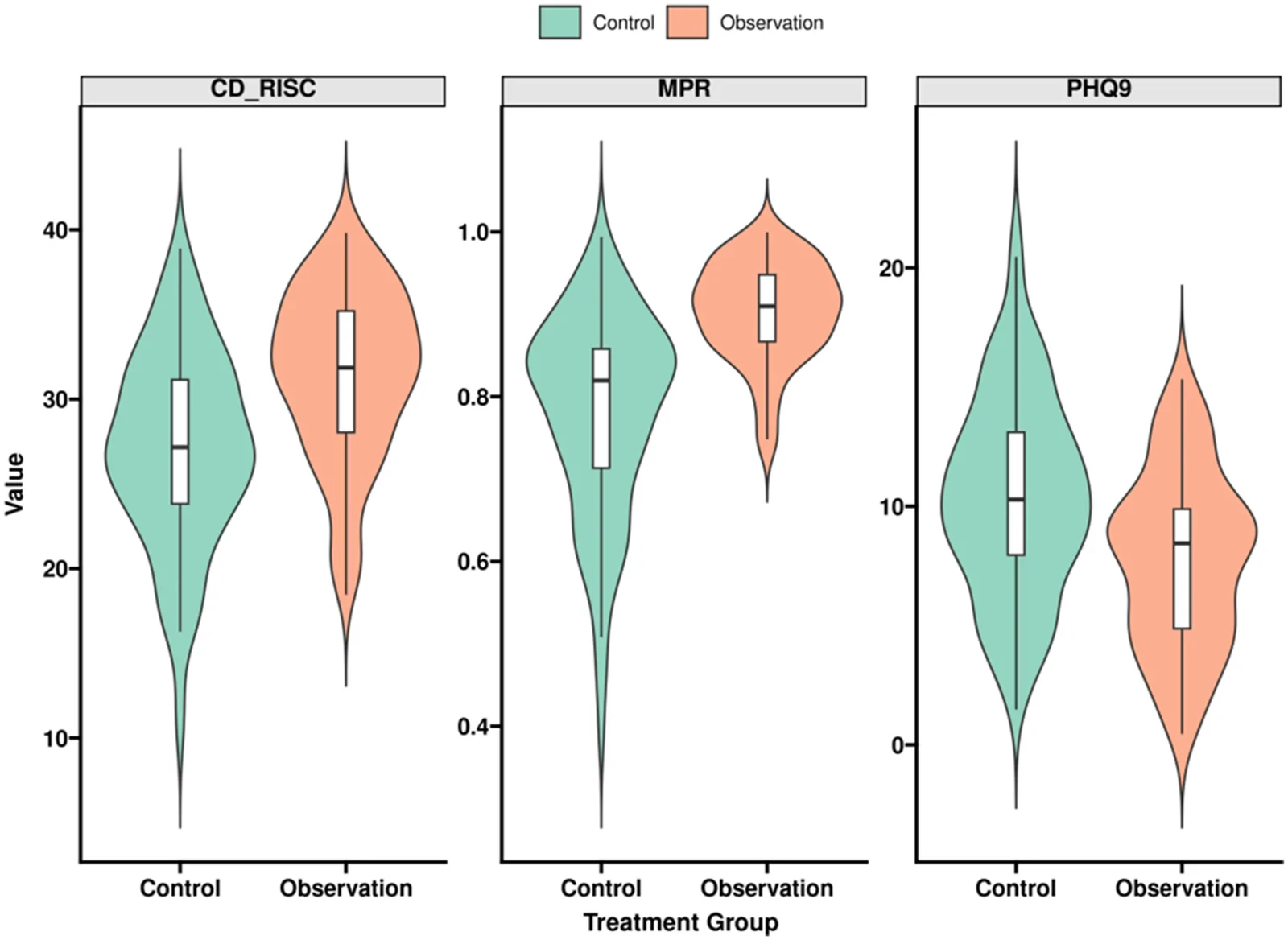
Comparison of primary psychological and behavioral outcomes at 6 months.

### 3.4 Comparison of virological and biochemical outcomes

Table 4 compares the virological and biochemical outcomes. The virological response rate was higher in the observation group (85.0% vs. 70.0%; *χ*^2^ = 6.45, *P* = 0.011). The ALT normalization rate was also higher in the observation group (80.0% vs. 65.0%; *χ*^2^ = 5.64, *P* = 0.018). The mean change in HBV DNA (log10 IU/mL) was greater in the observation group (t = 3.68, *P* < 0.001), as was the mean change in ALT (t = 3.74, *P* < 0.001). See Table 4. These unadjusted differences should be interpreted cautiously because allocation to the resilience program was voluntary and non-randomized; adjusted estimates are presented in Table 7 and Supplementary Table S2.

**Table 4:** Comparison of virological and biochemical outcomes.

Outcome measure	Observation group (*n* = 100)	Control group (*n* = 100)	Statistic	*P*-value
Virological response rate	85 (85.0%)	70 (70.0%)	χ^2^ = 6.45	0.011
ALT normalization rate	80 (80.0%)	65 (65.0%)	χ^2^ = 5.64	0.018
HBV DNA change (log10 IU/mL)	−3.45 ± 1.23	−2.78 ± 1.34	t = 3.68	< 0.001
ALT change (U/L)	−45.67 ± 23.45	−32.34 ± 26.78	t = 3.74	< 0.001

### 3.5 Quality of life and adverse events

Table 5 presents the health-related quality of life and treatment safety outcomes. The SF-36 Physical Component Summary (PCS) score was higher in the observation group (t = 3.96, *P* < 0.001). The SF-36 Mental Component Summary (MCS) score was also higher in the observation group (t = 5.99, *P* < 0.001). No difference was observed in the incidence of adverse events related to antiviral therapy between the two groups (*χ*^2^ = 0.33, *P* = 0.568). See Table 5.

**Table 5:** Quality of life and adverse events.

Outcome measure	Observation group (*n* = 100)	Control group (*n* = 100)	Statistic	*P*-value
SF-36 PCS score	75.67 ± 12.34	68.45 ± 13.45	t = 3.96	< 0.001
SF-36 MCS score	72.34 ± 11.23	62.34 ± 12.34	t = 5.99	< 0.001
Adverse event incidence	15 (15.0%)	18 (18.0%)	χ^2^ = 0.33	0.568

### 3.6 Comparison of healthcare utilization

Table 6 compares healthcare utilization during the 6-month follow-up period. The median number of unplanned outpatient visits was lower in the observation group (U = 4,181.5, *P* = 0.045). In a Poisson regression sensitivity analysis adjusting for the same covariates, the observation group had a lower rate of unplanned outpatient visits (adjusted rate ratio 0.72, 95% CI 0.56 to 0.93). The incidence of emergency department visits or hospital admissions did not differ between groups (*χ*^2^ = 2.41, *P* = 0.121). See Table 6.

**Table 6:** Comparison of healthcare utilization.

Outcome measure	Observation group (*n* = 100)	Control group (*n* = 100)	Statistic	*P*-value
Outpatient visits [M (IQR)]	2 (1–3)	3 (2–5)	U = 4,181.5	0.045
ED visit/Hospitalization events	8 (8.0%)	15 (15.0%)	χ^2^ = 2.41	0.121

### 3.7 Independent associations of resilience intervention on various outcomes

Table 7 summarizes the adjusted associations between receiving the resilience program and each outcome. All models were adjusted for age, sex, body mass index, baseline HBV DNA, baseline ALT, antiviral agent type, and presence of cirrhosis. Receiving the resilience program was associated with lower PHQ-9 scores (adjusted *β* = −3.20, 95% CI −4.50 to −1.80), higher MPR (adjusted *β* = 0.124, 95% CI 0.078 to 0.170), higher CD-RISC-10 scores (adjusted *β* = 4.12, 95% CI 2.79 to 5.46), higher odds of virological response (adjusted OR=2.46, 95% CI 1.35 to 4.48), higher odds of ALT normalization (adjusted OR=2.01, 95% CI 1.10 to 3.67), higher SF-36 MCS scores (adjusted *β* = 8.57, 95% CI 5.23 to 11.90), and lower odds of having ≥3 unplanned outpatient visits (adjusted OR=0.47, 95% CI 0.24 to 0.89). Because of the observational design, these are adjusted associations, not causal effects.

**Table 7:** Multivariable regression analysis: independent association of resilience intervention on outcomes.

Model	Dependent variable (outcome)	Predictor (resilience-based care vs. control)	Adjusted estimate (95% CI)	*P*-value
1	PHQ-9 score (Continuous)	Resilience-Based Care	*β* = −3.20 (−4.50, −1.80)	< 0.001
2	MPR (Continuous)	Resilience-Based Care	β = 0.124 (0.078, 0.170)	< 0.001
3	CD-RISC-10 score (Continuous)	Resilience-Based Care	β = 4.12 (2.79, 5.46)	< 0.001
4	Virological response (Yes/No)	Resilience-Based Care	OR=2.46 (1.35, 4.48)	0.003
5	ALT normalization (Yes/No)	Resilience-Based Care	OR=2.01 (1.10, 3.67)	0.023
6	SF-36 MCS score (Continuous)	Resilience-Based Care	β = 8.57 (5.23, 11.90)	< 0.001
7	Unplanned outpatient visits ≥3 (Yes/No)	Resilience-Based Care	OR=0.47 (0.24, 0.89)	0.021

In Model 7, the dependent variable “Unplanned outpatient visits ≥3” is a dichotomized outcome derived from the continuous variable, using the upper quartile of the control group (3 times) as the threshold to analyze the intervention's effect on reducing high healthcare resource use. All models were adjusted for covariates as described in the text. β: Beta coefficient, representing the mean difference in outcome scores between the intervention and control groups; OR: Odds ratio, representing the odds of achieving a positive outcome in the intervention group compared to the control group.

Adjusted baseline-to-follow-up change analyses using ANCOVA confirmed that the intervention group exhibited significantly greater reductions in PHQ-9 scores (adjusted mean difference −3.2, 95% CI −4.5 to −1.8, *P* < 0.001) and greater increases in MPR (adjusted mean difference 0.11, 95% CI 0.06–0.16) compared to controls, after accounting for baseline values (Supplementary Table S1∼S2).

## 4 Discussion

This retrospective cohort study examined the association between receiving a structured resilience-based program in addition to standard care and depressive symptoms and medication adherence in patients undergoing antiviral therapy for CHB. The results showed that patients in the observation group had more favorable scores across several psychological, behavioral, virological, and biochemical domains. However, these are adjusted associations from non-randomized data and should not be interpreted as evidence of causality. The consistency across domains suggests that further prospective evaluation of such programs may be warranted, but the findings are hypothesis-generating (15, 16).

Regarding psychological and behavioral outcomes, the observation group had lower depression scores, higher MPR, and higher resilience scores. These associations are biologically plausible but remain hypothetical. The program incorporated cognitive-behavioral techniques and positive psychology principles that may reduce disease-related automatic negative thoughts and improve self-management motivation, but these mechanisms were not directly measured. Skill-building components such as problem-solving and relaxation techniques may also facilitate behavioral change, but this interpretation is speculative. Previous studies have reported that similar psychological interventions are associated with reduced depression and improved adherence in chronic disease populations, although many focused on single outcomes. The present study extends these observations by examining multidimensional outcomes within CHB (17, 18).

For virological and biochemical outcomes, the observation group had higher virological response and ALT normalization rates and greater reductions in HBV DNA and ALT. A possible explanation is that better medication adherence may have contributed to these differences, but this was not formally tested as a mediation pathway. Neuroendocrine and autonomic mechanisms linking psychological distress to immune control of HBV are plausible hypotheses, but were not evaluated because biomarkers such as cortisol and cytokines were not measured. Prior observational studies have reported associations between psychological factors and virological outcomes in HIV and HCV, but evidence in CHB remains limited. These findings should therefore be viewed as generating hypotheses for future mechanistic work (19, 20).

Quality of life scores were higher in the observation group, and the incidence of adverse events was similar between groups. Although no safety concerns were observed, the statement that the program “can safely enhance” well-being would be too strong for an observational study. The observed difference in quality of life may reflect better emotional and functional adaptation, but this remains an association. Previous literature has reported improved quality of life after psychological interventions in cancer and cardiovascular disease; the present findings are consistent with those reports but require prospective confirmation in CHB (21, 22).

The observation group had fewer unplanned outpatient visits, although the difference in emergency department visits or hospital admissions was not statistically significant. This may reflect better symptom management or reduced distress-driven healthcare seeking, but these explanations are speculative. The possibility that such programs could reduce healthcare utilization deserves further health-economic evaluation, but the present data are insufficient to support a cost-containment claim (23, 24).

Multivariable regression analysis showed that receiving the resilience program was associated with more favorable outcomes after adjustment for measured confounders. However, these are adjusted associations, not independent effects. Residual confounding by unmeasured factors such as baseline motivation, social support, and personality traits cannot be excluded, and the retrospective design precludes causal inference. Future prospective randomized studies are needed to determine whether the observed associations reflect a true intervention effect or selection bias (25, 26).

This study has several important limitations that should be carefully considered when interpreting the findings. First, the retrospective, non-randomized design and voluntary participation in the resilience program introduce substantial self-selection bias. Patients who chose to attend the intervention may have had higher intrinsic motivation, better social support, more adaptive personality traits, and a greater baseline propensity for adherence—characteristics that cannot be fully captured by measured covariates. Consequently, despite multivariable adjustment, residual confounding may substantially inflate the observed benefits, and no causal conclusions can be drawn. Second, the absence of randomization and the lack of propensity score matching or inverse probability weighting (not performed because of the modest sample size and the risk of model overfitting) further limit the ability to balance unmeasured confounders between groups. Third, the follow-up duration was only 6 months, which is insufficient to assess the sustainability of any psychological, behavioral, or virological improvements or to capture long-term endpoints such as HBsAg seroclearance or liver-related complications. Fourth, key hepatological covariates were incompletely captured: quantitative HBsAg levels, fibrosis stage assessed by biopsy or transient elastography, detailed treatment switch histories, and concurrent metabolic comorbidities such as non-alcoholic fatty liver disease were not systematically recorded, which may introduce additional residual confounding. Fifth, uptake of external mental health care (e.g., private counseling, antidepressant pharmacotherapy) during follow-up was not monitored and could have influenced depression and adherence trajectories. Sixth, the study was conducted in a single Chinese center, and the sociodemographic and cultural characteristics of this cohort limit the generalizability of the findings to other populations and healthcare settings. Finally, multiple correlated outcomes were tested without multiplicity adjustment, increasing the risk of type I error, and given the sample size the regression models may be overfitted, so the reported effect estimates and confidence intervals should be interpreted with caution. These limitations underscore the need for well-designed randomized controlled trials with longer follow-up, more comprehensive covariate measurement, and ideally multicenter recruitment to confirm or refute these exploratory findings.

In conclusion, this retrospective study suggests that a structured resilience program is associated with more favorable psychological, behavioral, and clinical outcomes in CHB patients. Given the observational design and substantial risk of selection bias, these findings are hypothesis-generating and should not be interpreted as evidence of efficacy. Well-designed randomized controlled trials with long-term follow-up and biomarker assessments are needed before considering integration into routine care.

## Data Availability

The original contributions presented in the study are included in the article/supplementary material, further inquiries can be directed to the corresponding author.
