# Supplementary material for: Depression and medication adherence among chronic hepatitis B patients receiving resilience-based care versus standard care during antiviral therapy: a retrospective cohort study
Source: Front Med (Lausanne). 2026 Sep 21;13:1832041. doi: 10.3389/fmed.2026.1832041 (PMC13634855; doi:10.3389/fmed.2026.1832041)
Supplement: Supplementary file 1 [file Table1.docx]

Supplementary Table S1. Adjusted baseline-to-follow-up changes in continuous outcomes (ANCOVA models)

| Outcome variable | Group | Baseline | Follow-up | Adjusted LS mean change (95% CI)* | Adjusted between-group difference (95% CI) | P value |
| --- | --- | --- | --- | --- | --- | --- |
| PHQ-9 score | Observation | 12.34 ± 4.56 | 6.78 ± 3.45 | –5.23 (–6.18, –4.28) | –3.20 (–4.50, –1.80) | <0.001 |
|  | Control | 11.89 ± 4.78 | 10.23 ± 4.12 | –2.03 (–2.98, –1.08) | Reference |  |
| CD-RISC-10 score | Observation | 23.45 ± 6.78 | 32.45 ± 5.67 | 8.67 (7.51, 9.83) | 4.12 (2.79, 5.46) | <0.001 |
|  | Control | 22.89 ± 7.12 | 26.78 ± 6.34 | 4.55 (3.39, 5.71) | Reference |  |
| MPR at 6 months^#^ | Observation | – | 0.92 ± 0.08 | 0.91 (0.88, 0.94) | 0.124 (0.078, 0.170) | <0.001 |
|  | Control | – | 0.78 ± 0.15 | 0.79 (0.76, 0.82) | Reference |  |
| HBV DNA change (log₁₀ IU/mL) | Observation | 5.67 ± 1.23 | – | –3.35 (–3.61, –3.09) | –0.52 (–0.90, –0.14) | 0.008 |
|  | Control | 5.54 ± 1.34 | – | –2.83 (–3.09, –2.57) | Reference |  |
| ALT change (U/L) | Observation | 68.45 ± 45.67 | – | –42.8 (–49.5, –36.1) | –10.5 (–18.2, –2.8) | 0.007 |
|  | Control | 72.34 ± 48.23 | – | –32.3 (–39.0, –25.6) | Reference |  |

^*^Least-squares (LS) means and 95% confidence intervals were estimated from analysis of covariance (ANCOVA) models adjusted for age, sex, body mass index, baseline HBV DNA, baseline ALT, antiviral agent type, and presence of cirrhosis.

^#^ MPR (Medication Possession Ratio) was calculated over the 6-month follow-up period; no baseline MPR exists. The adjusted between-group difference therefore represents the covariate-adjusted difference in MPR at 6 months.
