# Supplementary material for: Depression and medication adherence among chronic hepatitis B patients receiving resilience-based care versus standard care during antiviral therapy: a retrospective cohort study
Source: Front Med (Lausanne). 2026 Sep 21;13:1832041. doi: 10.3389/fmed.2026.1832041 (PMC13634855; doi:10.3389/fmed.2026.1832041)
Supplement: Supplementary file 2 [file Table2.docx]

Supplementary Table S2. Adjusted odds ratios for binary clinical outcomes (multivariable logistic regression)

| Outcome variable | Observation group n/N (%) | Control group n/N (%) | Adjusted OR (95% CI)* | P value |
| --- | --- | --- | --- | --- |
| Virological response (HBV DNA <20 IU/mL) | 85/100 (85.0%) | 70/100 (70.0%) | 2.46 (1.35, 4.48) | 0.003 |
| ALT normalization (≤35 U/L men, ≤25 U/L women) | 80/100 (80.0%) | 65/100 (65.0%) | 2.01 (1.10, 3.67) | 0.023 |

* Odds ratios were adjusted for age, sex, body mass index, baseline HBV DNA, baseline ALT, antiviral agent type, and presence of cirrhosis. OR >1 indicates higher odds of achieving the outcome in the observation group compared with the control group.
